# Pathological Society of Philadelphia

**Published:** 1881-02

**Authors:** 


### Reports of Societies.

---

### PATHOLOGICAL SOCIETY OF PHILADELPHIA.

*Aneurism of the cœliac axis.*—Presented by Dr. E. T. Bruin—These specimens were removed from the body of a colored man, aged about 32, who died in the Philadelphia

Hospital, a few weeks since. In abstracting my notes on his case I recorded that a syphilitic history was admitted, but that the individual had been a temperate man in the use of alcoholic drinks.

A pulsation could be felt about two and a half inches below the ensiform cartilage, but a distinct, tumor could not be felt. There was no thrill. Careful percussion developed impaired resonance in area the breadth of the m ddle finger only. By auscultation, a murmer very prolonged, low-pitched, and of systolic rhythm. There was no bruit, nor could the murmer be heard in the femorals or at the bifurcation of the iliacs ; it could not be heard at the back. It was heard first at ensiform cartilage, grew louder till a point two and a half to three inches below the sternum was reached, and then faded away. A deep seated burning pain was lamented, and also a scattering pain radiating around to the anterior median line along the ribs: this pain he described as sharp, and at times it was much worse than others. Both sorts of pain were relieved by change of posture. His favorite attitude was lying prone on the face. This helped vastly to mitigate the pain.

A treatment of rest was commenced, and it was designed to place upon him a plaster jacket, commencing the roller high up in the chest and to continue it to the hips. It was hoped that this would aid in fixing the aorta and aid in the treatment of rest in bed, a restricted and selected diet, and the drugs usually employed in these cases. It was decided to apply this dressing on a Wednesday. The day before, the man rose in the morning, expressed himself as feeling quite well, and was engaged in making his toilet, when he suddenly cried out and dropped dead.

*Autopsy*.—The abdominal cavity was filled with coagulated blood. A saccular aneurism about as large as a good sized lemon projected from the left antero-lateral surface of the aorta. The walls of the sac were most attenuated; at the periphery of the tumor the external coat was about as thick as a piece of paper, and very brittle from calcareous deposit. The sac, when laid open, was found to be only partially filled with friable clots, not organized into layers, but irregularly disposed over its walls. The rupture occured at the point most remote from the aorta, at the periphery of the tumor. The orifice communicating with the aorta was very small, about the size of a large hickory-nut. The other parts of the aorta and the heart were normal. The lungs, liver and kidneys presented a natural appearance.

I would comment briefly on the infinite importance of quiet and rest in the treatment, and the advantage ol fixing the aorta by means of the permanent dressing. Certainly the lateral movements of the body would have been much restricted, and the aorta probaly saved many a flexion. Only at the last meeting I spoke of the supreme importance to the diagnostician of the prior history of the conditions favoring arteritis, and the evidence of pressure in the path of the aorta, and percussion-signs of tumor. In addition to what I then said, let me add a word concerning the murmur as present in the case. The absence of bruit, the prolonged low-pitched murmur, with the systole, suggested to me that this was a fusiform aneurism caused by a general dilatation of all the coats of the aorta, especially as the evidence of tumor was so slight so far as percussion was concerned.

The orifice communicating with the aneurism was so small that very little blood probably entered and left the sac; besides, the sac was partially filled with clot, and at the autopsy it lay on the trunk of the vessel, doubtless obstructing its lumen, and I suggest it may have caused the murmur. The ante mortem inference as to the shape of the aneurism has been, to my mind, sustained by the autopsy.

I show with this case an aneurism exibited some time ago to this society, the sacs entirely filled with laminated clot, and in which case there was no murmur, unless the tumor which projects anteriorly at the second right costal cartilage, was pressed upon so that the calibre of the aorta was lessened. A distant, long, low-pitched systolic murmur, without a taint of bruit, indicates to me that the sac of an acurism is mostly filled with clots; and, unless I can identify a tumor by palpation or percussion, or by the symptoms of pressure on adjacent organs, I am apt to infer the existence of an aneurism by general dilatation of the coats of the vessel from the above mentioned murmur. I do not mean to say that these general dilatations do not cause pressure-symptoms, but merely that these are not so pronounced, as a rule. But in this case the murmur deceived me, and a saccular aneurism only half filled with clots exitsts; but I hope the very small opening leading into the aorta will be deemed a valid reason for the absence of pronounced bruit.

Dr. Nancrede took exception to the term "abdominal" aneurism, as he thought that a careful examination of the specimen showed conclusively that it was due to a dilatation of the cœliac axis, and did not involve the aorta at all.

Dr. Bruen thought that this trunk might be partly involved, but was inclined to think that a part of the walls of the aneurism was formed by the aorta.

Dr. Nancrede explained the anatomical reasons upon which he had founded his opinion, and which he thought were conclusive.

The President said that, as there seemed a difference of opinion on the subject, he would refer the specimen for examination to a special committee consisting of Drs. Bruen, Nancrede, and Formad.

"The committee appointed to examine the tumor agree to pronounce it an aneurism of the cœliac axis rather than aorta proper. This explains the fact that the orifice leading into the aneurism is so small, as it is the dilated cœliac vessel; but remarks in reference to the etiology of the murmur are equally pertinent.

EDWARD T. BRUEN,
C. B. NANCREDE,
H. F. FORMAD."

*Spindle celled Sarcoma of the Skin.*—Presented by Samuel W. Gross, M.D.—Records of examples of sarcoma of the skin in the medical journals and references to that disease in the text-books are so very uncommon that I am induced to bring the specimen before the Society.

A married woman, 23 years of age, was brought to my clinic at the Jefferson Medical College Hospital on the 29th of September, 1880, on account of a painless tumor of the integument of the buttock, which was of the size of a small lemon, of a firm elastic consistence, and with a discolored and rugous surface. She first noticed it as a shot like tubercle about three years previously, and she informed me that she had struck it a few weeks before, and that the injury was followed by slight bleeding and rapidity of growth.

On a section after removal, the cut surfaces were of a rosacerous yellow shining hue, and had a fibrous tear. Microscopically, the growth was characterized, as can be seen from the accompanying slide, by small spindle cells contained in a homogeneous intercelluar substance. Up to the present time the tumor has not recurred.

Dr. Duhring asked whether in this case there was more than one growth. Although it was now, of course, difficult, or impossible, to determine the exact starting-point, of the neoplasm, yet he was inclined to think that its site had been the subcutaneous cellular tissue. Dr. Gross, he remarked, was correct as to the rarity of such growths, all authorities making similar statements. Indeed, Kaposi, the most recent German writer, one who has devoted especial attention to the pathology of skin affections, only devotes a short chapter in his treatise to sarcoma of the skin. He treats of the disseminated pigmented form only, which seems to be the most usual multiple form assumed by sarcoma when attacking the integument. Dr. Duhring knew of only one instance which had been reported in this country within the past ten years. This variety of sarcoma, however, was of such a different form from that presented this evening that he would not enter into any further details.

The whole subject of sarcoma of the skin had been opened up by a case reported by him. He had described the case at the meeting of the American Dermatological Association held at Saratoga in 1878 as an "Inflammatory Fungoid Neoplasm." A year later, at the third meeting of the same association, he had read a supplemental report upon the case, when, owing to the report of Dr. Heitzmann, he had been inclined to suspect its sarcomatous nature. The disease represented by this case was closely allied to sarcoma, if not true sarcoma. About the same time Geber reported a similar case, also under the caption 'Inflammatory Fungoid Neoplasm."

Kaposi, in commenting recently on these cases, says that he has no doubt of their sarcomatous nature. All the cases so far have proved fatal in about two or three years. Dr. Gross had described his case as a "spindle celled" sarcoma. Dr. Duhring would like to ask whether in his experience, the round cell was not much the commoner variety.

Dr. Gross said that he had notes of eight cases of sarcoma of the skin, but that he had been unable to find them in time for the meeting. He was, however, of the impression that Dr. Duhring's statement as to the greater frequency of the round-celled form was correct. The general history of these neoplasms is that they are most common after the fortieth year, that they soon ulcerate, and that they exhibit a decided tendency to recur after removal. With regard to metastases and the final termination he was quite in the dark, as he had been unable to follow his cases. He thought that the apparent rarity of the disease was explicable by the fact that tumors usually come under the care of surgeons, and not of dermatologists. He would call Dr. Duhrings attention to Billroth's "Chirurgische Klinik," in the several volumes of which publication about twenty cases of sarcoma of the skin of various portions of the body are detailed.

Dr. Duhring called in question the propriety of the term "sarcoma of skin," and thought that its primary seat had been the subcutaneous connective tissue.

Dr. Gross said that there could be no doubt that it arose from and was limited to the skin, and stated the reasons for his belief.

Dr. Formad said that he had lately examined one of many nodules situated in the skin, which had been removed and sent to him by Dr. Nash, of Norfolk, Va. The microscope demonstated that the growths consisted of round-celled sarcomatous tissue originating in the true skin.

*Cancer of liver, with metastasis to the brain and other organs —Destruction of the occipito-temporal convolution without affection of special sense.*—Exhibited by Dr. H. F. Formad for Dr. H. C. Wood —T. C., æt. 42, came to the nervous wards of the Philadelphia Hospital early in September, 1880. He stated that he had been a heavy drinker, but had enjoyed good health until the lasl of August. On September 2 he was seized with giddiness, which lasted over twenty-four hours, but which did not leave him paralyzed. He affirmed that this was the first spell of the sort he had ever had, and denied syphilis, headache, or any other known cause or premonitory syptom of the attack. Since the period of unconsciousness he has suffered from continued headache, loss of memory, giddiness, and frequent vomiting, the latter occuring without connection with the state of the stomach as to food, and not rarely in the night, as well as in the daytime.

Reptember 15.—There was no signs of local palsies, and no disorder of vision, and the gait was perfect. There was partial bilateral deafness, which he states was of long standing; there was no anæsthesia. No disorder of the taste or smell was detected, although neither was closely looked for. The right hand exerted a power of 85, the left hand of 92. The liver was found to be very much enlarged, hard and nodular, and the diagnosis of hepatic cancer was reached. There was no changes in his nervous symptoms, and he was sent to the general medical ward, October 27.

During the last two weeks of his life his mental powers failed very distinctly, his speech became somewhat incoherent, and his memory exceedingly faulty. He died November 10.

*Autopsy.*—Brain normal, except the under central portion of the posterior lobe of the left side; springing from the membranes in this position was an irregularly ovoid tumor about an inch and a half in the greatest longitudinal and transverse diameters, and about an inch thick. The tumor was of elastic, hard consistence, of dark red color, and had the appearance of a firm old blood-clot; it was not encapsuled. It had pressed upon and destroyed, by softening, almost the whole of the central portions of the occipito-temporal convolutions. The hippocampus was touched upon, but not altered to any extent. The softening had progressed so far as to open the lateral ventricle, the destruction not involving any of the central ganglia or the crus cerebri.

The membranes of the brain were abnormally thickened everywhere, and the fissures were bound together by inflammatory exudations. The one corner of the tumor had produced a small superficial spot of softening in the cerebellum; the attachments of the tumor to the surrounding tissues were very loose. A decided increase of cerebral fluid, turbid in appearance, was observed. Skull normal.

Examination of the thoracic cavity revealed pericardium and right lung and pleural sac normal. In left pleural cavity extensive adhesions and two pints of a turbid effusion were found; left lung strongly congested, and a wedge-shaped cavity of evidently embolic origin was seen.

Mediastinal and bronchial glands enlarged; heart normal. The abdominal cavity contained over one gallon of a straw colored liquid; no peritonitis.

Liver very much enlarged; weight nine and a half pounds, of dark-brown color, and studded with the protruding, umbilicated, white nodes peculiar to cancer, varying in size from one-half inch to five inches in diameter. The head of the pancreas was also involved by similar nodes. Mesenteric glands greatly enlarged and noded. Kidneys invaded by a number of small nodules, scattered throughout the capsules as well as the parenchyma of the organs.

Stomach, intestines, rectum, and rest of organs normal.

*Microscopic examination* by Dr. H. F. Formad determined the structure of the new growth of the liver, of the brain, and of the other organs to be that of a *cylindrical* epithelioma, the primary growth having developed in the liver.

Dr. Formad added that the new growth, of the brain especially, showed typically the peculiar cones built up of cylindrical epithelium piercing through a vascular connecting tissue in every direction; that at the same time, however, it demonstrated in places well the direct transformation of a cylindrical epithelioma into a simple glandular carcinoma, as described first by Perls, the solid cones of the epithelioma breaking up; and the cylindrical epithelial cells are distinctly seen *transforming* into spheroidal cells, which fill, loosely packed, the alveolar spaces.

*Tuberculous Growth* (*tyroma*) *of Cerebellum.*—Exhibited by Dr. H. F. Formad.—This specimen and some notes of the history I obtained through the kindness of Dr. E. R. Girvin, who will present the interesting history of the case in full to the County Medical Society.

Miss G., aged 21, was never confined to bed with any sickness prior to the malady which terminated fatally. She complained, however, of occasional headache during the last eleven years, which she always located at the occipital region. She received an injury eleven years ago by a fall from a swing, striking this region of the head upon a projecting board, the accident being followed by loss of consciousness, which lasted, however, only a few minutes. With the exception of the periodical localized headache, which started from the time of the injury, she remained perfectly well.

In February, 1880, the parozysmal headache became of more grave nature, the attacks developed in frequency to one every five or six days, each attack lasting three to four or six days, the pain radiating over the entire top of the cerebrum, and attended with great hyperæsthesia. The skin cool; pulse compressible, thready and irregular, ranging from 70 to 100. During April, May and June the patient improved, but subsequently the attacks recurred with increased severity, and were followed by difficulty in vision—viz: double sight. Examination by Prof. W. F. Norris revealed choked disks affecting both eyes. Later, complete blindness ensued. There was a slight loss of power of left side of body, and sometimes twitching of the muscles of left side of face was noticed and staggering gait. She complained sometimes of a momentary swinging or rotary sensation. The intellect was clear and the memory not impaired. During August and September the condition of the patient again improved. September 27 the attack suddenly became more severe, and, growing in intensity, terminated by death on October 1. During the last few days of life there was loss of power of deglutition, also of sense of smell, taste and hearing; general sensibility and clearness of mental faculty were perfect up to death.

A complete autopsy was objected to. I examined only the brain, with the following result:

Skull normal. Dura mater normal, except over the left side of the cerebellum, where it was thickened and adherent to the bone. Pia mater opaque, slightly thickened, and showing evidence of previous strong congestions and repeated effusions.

Examining the cerebellum by section, there were seen a number of yellowish-gray nodes of a new growth, varying in size from one-fourth to three-fourths of an inch in diameter, located both in the gray and white matter of the left side and at the middle of the cerebellum, in close proximity with, but not involving, the pia mater. The left side of the cerebellum was also thicker than the right. Over the seat of the tumefaction was a distinct localized septomeningitis, and adhesions between the membranes.

The cerebrum was very anæmic, otherwise of perfectly normal appearance in all parts. An increased quantity of fluid was noticed in the lateral ventricles.

*Microscopic examination.*—Nothing abnormal in cerebrum; the pia mater showed, however, numerous tubercle granulations in the course of the blood vessels. Examination of the new growth of the cerebellum proved it to be of a tuberculous nature, showing the structure of a nonvascular granulation tissue containing numerous nodules, some of which were undergoing cheesy degeneration. Transverse sections of obliterated blood vessels, and also true giant cells, were conspicuous. The granulations of the periphery of the nodes show a distinct attempt at fibrillation—the formation of a capsule. The location also of the new growth of such structural elements leaves the diagnosis of *tyroma* beyond doubt, excluding two other possible new formations, the gumma and glioma.

The direct etiological relation of the new growth to the injury received eleven years prior to death is here highly probable, and hence in favor of the theory of inflammatory origin of tumors.

*Cancer of Stomach*.—Presented by Dr. J. H. Musser—The patient whose stomach I present for your examination applied to me for treatment the 11th of March of the present year. From him I gleaned the following history:

H. O., æt. 55; butcher; moderate drinker; excessive smoker; has been a hard worker; unfortunate in business, and of late years in poor circumstances; has had considerable mental strain; always healthy until present illness; no venereal diseases; family history good; wife died of cancer of the stomach eight months ago.

Present illness was of six months' duration. Increasing languor, debility, and emaciation, dyspnœa on exertion, continual yawning, sleeplessness and loss of memory have been noticeable symptoms. What distressed him, however, was a weight and fullness after meals in the stomach, with flatulence, lasting two or three hours, and only being relieved by forced vomiting; thirst has been very great; constipation persistent and increasing.

His condition when examined was as follows. Large, heavily framed; very much emaciated, having lost seventy pounds; countenance did not indicate suffering from pain, but from mental torture; face emaciated; eyes sunken; conjunctiva pale; sclerotic dead-white; lips pale; complexion dirty yellow; extremities cold; speech slow sighs considerably; mind is sluggish; memory poor; heart and lungs normal; pulse 90, feeble; tongue large, pale, flabby, heavily coated; teeth poor; throat dry, slight pharyngitis; appetite fair; thirst great; after eating, weight and fulness in the stomach; great flatulence; never vomits involuntarily; when he provokes vomiting the matters ejected are the contents of the stomach and greenish fluid, never blood; bowels very costive.

*Inspection* of abdomen reveals a pulsation between the xiphoid cartilage and the umbilicus, and a distinct prominence to the right of the median line above the umbilicus. *Palpation* defines the bulging to be two and a half inches above the umbilicus, a little to the right of the median line, and extending within oue half inch of the ribs. *Percussion* does not define any resonance between the normal dulness of the liver's margin and the mass. No pain on pressure, and no increase of local heat; no murmur on auscultation. Liver and spleen normal size; urine not examined.

May 1.—Learned from one of his family that he had emaciated very much and become much debilitated.

May 24.—Called to him again and found him extremely emaciated. Abdomen very scaphoid. Pulsation very visible. Mass observed two inches above umbilicus, between the median line and the ribs. Percussion defines the tumor in that area, extending three inches along the ribs. The size of the mass is greater than when first examined, or the scaphoid state of the abdomen causes it to be more readily outlined. No tenderness on pressure. Paroxysmal pain of a burning or sharp, shooting character Almost constant vomiting. Matters vomited are the food and an acid, white liquid, burning the fauces and mouth At times, however, free from vomiting, so that to relieve the distressing sense of fulness he has to provoke it. Tongue large, pale, flabby, coated; pulse 96; bowels constipated. Skin itchy, dry, with a general pustular eruption on it. No sleep; very restless.

May 26.—A powder, given every three hours, containing morph. sulph., gr. 1-12 sodæ bicarb, lactopeptine, aa gr. ij, pulv. aromat., gr. 1-12, prescribed on the 24th, gave relief to the pain, stopped the vomiting, caused sleep, and permitted the more liberal use of milk.

The symptoms were all better, though he took scarcely any nourishment, and on the 31st of the month he died of exhaustion.

*Post-mortem* (twenty-four hours after death).—Body extremly emaciated; abdomen concave; skin of same color as before death, very rough; rigor mortis well marked.

Abdominal cavity alone examined. Tissues bloodless on section; organs in normal position; no ascites.

*Stomach* very much dilated; vessels congested, both arteries and veins, especially near the pylorus. It contained a dark-gray liquid; no blood. At the cardiac end of the stomach the mucous membrane was very pale, becoming more congested towards the pylorus, with swelling and softening of the mucous membrane and some thickening of the walls towards the pylorus. At the pylorus the walls were much thickened, from one-half to three-fourths of an inch, and projected into the stomach as a shelf. The walls of the duodenum were involved to the extent of two and a half inches from the pylorus. They were infiltrated by a dense, firm mass of tissue, softer an inch from the pylorus than at the pylorus. At some points the thickening was greater, giving the appearance of nodules. Neither the pyloric orifice nor the intestine was occluded, both admitting one finger. I neglected to state that the congested portion of the mucous membrane was bathed with mucus. The mesenteric glands were enlarged.

The *liver* was slightly fatty; weighed two pounds fifteen ounces. The other abdominal organs were natural to a person dying of chronic disease. The pancreas was normal, situated back of the mass, and removed with it.

*Remarks.*—At the time of the first examination cancer was diagnosed, and an unfavorable prognosis given. I was not called in again until shortly before death; hence the absence of notes in the interval. The symptoms and course were characterlstic of cancer of the stomach, and the autopsy confirmed the opinion. The microscopical appearances of the tumor are those of a scirrhus, with a few spots of degeneration.

*Cirrhosis of the Liver and Kidneys—Liver deformed—Death from Softening of the Brain.*—Presented by Dr. J. W. Musser—I visited the patient from whom the specimens before you were taken the first time on the 8th of October, 1879. Her condition at that time and the subsequent course of the disease are embodied in the following notes:

Mrs. H., æt 57, born in Ireland, a resident of Philadelphia, for the past two years living in an intensely malarial district near the Schuylkill. Different members of the family have had malaria the past three months. Married. Eleven children, six living and healthy; the others died of acute disease in early childhood or youth. Circumstances and comforts of life quite moderate. Considerable mental worry from the cares of a large family and on account of husband's intemperance. Habits good.

Father died of "jaundice." Cause of mother's death unknown. A sister died of consumption. No further evidence of hereditary disease.

Always healthy, save an attack of ague twenty years ago, and, for several years, frequent attacks of "billiousness." These attacks were characterized by anorexia, nausea, and vomiting of a yellow-and-green matter, with or without diarrhœa. No syphilis.

For six months patient has suffered from poor appetite, bad taste in the mouth, flatulency and acidity, and irregular bowels. The past three weeks has been quite miserable, being without appetite and very weak. Cramp-like pains, coldness and heaviness of the lower limbs have troubled her for a week, while for two days she has been vomiting.

At present (October 8,) she is quite weak, somewhat emaciated; extremities cold; skin of trunk warm and harsh, of a dark, muddy color; face of same color, with injected capillaries of the cheek; conjunctiva yellowish; heart normal; pulse 130, weak; lungs normal; breathing hurried, thirty to the minute. The hurried pulse and respirations are due to the exhaustion from vomiting. Nausea is constant; vomiting occurs every half hour. The matter vomited is a yellowish, sour liquid, with flakes of mucus in it; no blood. Sourness over stomach, but no pain.

Inspection of abdomen reveals a slight general enlargement, with localized bulging in the right hypochondrium. No enlarged veins. On palpitation, it is found that a smooth hard tumor occupies the following area: Extending from the outer border of the right rectus muscle to the axillary line. The lower margin is, at the outer border of the right rectus, one inch above a horizontal line through the umbilicus from left to right. In the nipple-line it extends to the line, in the axillary region to an inch above the crest of the ilium. No pain attends its handling. The upper boundary of the liver begins at the

Page(s) missing fifth rib and deep, in the sixth interspace on superficial, percussion. From thence dullness extends to the margin defined by palpation, being continuous with the tumor-dullness. The left hepatic lobe is not enlarged. Normal liver-dullness not increased posteriorly. With inspiration and expiration the entire mass moves, so that it is, with the evidence of percussion, connected with the liver. Spleen is not enlarged. Bowels are costive.

Appropriate diet and sedatives soon allayed the gastric symptoms, and in three days she was ordered cinchona, strychnia, and muriatic acid, and a regulated diet.

About three weeks afterwards her urine burned her very much when passed. It was found to be of an acid reaction, and a dark-red color, without albumen or sugar, containing urates and bile-pigment

In November she had a sudden, profuse, painless uterine hemorrhage. Her menses had ceased years ago. This was the only hemorrhage throughout her illness.

I saw her again December 11. She had emaciated somewhat. She was very weak. Her eyes were jaundiced, the capillaries of the face congested, and the skin of the face dark or bronzed, as previously noted. She had some slight fever, and a pulse of 110. Her appetite was poor, tongue coated brownish red, and glazed at the edges, thirst great, bowels irregular. Would not allow an abdominal examination. Suffers from pain in the loins. Urine is scanty. A dry, hacking cough annoys her very much. At times a dark-yellowish expectoration. No physical signs of bronchitis. Sight œdema of feet.

12th—Œdema subsided somewhat. Nausea and vomiting all night. Vomited matter at first like the yolk of an egg, later a watery fluid with flashes of green-tinged mucus floating in it, and green masses at the bottom of the vessel.

13th.—Vomiting better. No fever. Pulse 100. Liver as before noted; descends three inches, and is pushed to the left by the ilium. No œdema. Colic and diarrhœa last night. Much stronger. Urged continuance of the previously ordered acid tonic.

23d.—Another gastric attack, as previously.

January 16, 1880.—Noted as follows : Been visiting her every week, and find mind gradually failing. Wanders readily from a subject, talks of unreal occurrences, and of nonsensical matters. No memory. Sleeps poorly. Slight headache. Urine scanty, very red; specific gravity 1020; slight albumen. No jaundice. Considerable tympanitis.

21st.—Urine high-colored, acid; slight amount of albumen; no sugar; bile-salts and pigment. Microscopically, bladder-epithelium, pus, uric acid, and an abundance of phosphates. Growing weaker and weaker. Dry, brown tongue; slight gastric pain; no vomiting; regular bowels; slight ascites; no headache. Poor sleep; wandering delirium; refers to imaginary occurrences. Is continually enacting the movement of winding a spool of thread.

31st.—Peritoneal fluid increased. Pain in abdomen. Temperature not elevated. Pulse 120. "Some smothering about the heart." Mind less composed than before. Urine scanty.

February 2, 1880.—Urine scanty; eight ounces passed in twenty-four hours; high-colored. Microscopically, red blood-cells in abundance, a few white cells, epithelium from kidneys, ureters, bladder, and vagina; a few crystals of oxalate of lime. Urates, phosphates and tube-casts absent; bile-pigment in abundance. Chemically, bile, one-sixteenth albumen; no sugar.

5th.—Ascites increased. Tongue flabby, red and fissured. Vomited once,—bile. Bowels regular, fæces dark-colored. Pulse 120. No œdema of feet. Feeble; more stupid. Recognizes with difficulty, and comprehends poorly. At apex of heart a "murmurish" sound.

March 2.—Vomiting a dirty-yellowish, offensive, copious matter. Diarrhœa past two days; stools thin, clayey, offensive. Urine passed involuntarily. Is semi-conscious; aroused with difficulty, and murmurs unintelligibly. Lies on the right side, with the knees and legs drawn up and head bent to the chest; lack of co-ordination in movements for a week. Past three weeks face has had the appearance of dementia, as in cerebral softening; not the cirrhotic countenance.

12th.—Pulse 100, moderately strong, regular; respiration 32, short. Twitching of muscles of limbs. Can be aroused. Some hyperæthesia ; slight fever. Does not take food; urine and fæces passed involuntarily.

Became more and more unconscious, and died on the 13th in coma.

*Autopsy.*—Examined twenty-four hours after death, Dr. W. E. Hughes assisting.

Rigor mortis not well marked; body not extremely emaciated; skin dark, dirty color; no anasarca or jaundice.

Abdominal walls one and a half inches thick; fat moderately abundant. Cavity of abdomen almost filled with a clear, light straw-colored fluid; no flakes of lymph, and no adhesions; no congestion of the visceral or parietal peritoneum. Liver not displaced, occupied the position defined during life; other organs in normal positions. The liver was not nodular, but its surface was granular, and on section was dense and firm, granular with increase of connective tissue. A slight indentation marked this. The right lobe measured five inches from right to left, the left three inches. From above downwards the right lobe measured seven and a half inches, the left three inches. The right lobe was two and a quarter inches thick, the left two inches. It weighed two pounds two and a half ounces. Spleen normal in size, dense. Kidneys enlarged, congested, weighed ten ounces each; capsule easily removed; proportion between cortical and medullary portion normal.. Mucous membrane of stomach and intestines pale.

Heart, left ventricle contracted; mitral valve slightly thickened. Lungs slightly œdematous. Brain not examined.

Fluid of peritoneum contained albumen, sugar and urea. Kidneys, microscopically, were congested, and in early stages of cirrhosis. Liver markedly cirrhotic.

*Remarks.*—The cause of the enlargement only of the right lobe of the liver, and only of the anterior portion, at first puzzled me. It could not be a hydatid, or a purulent or serous collection between the liver and the diaphragm pushing the organ down, for the lungs were not encroached upon, nor were there any other confirming symptoms of such conditions, as fluctuation or fever. There were no symptoms or signs of disease of surrounding organs, as the kidneys, to cause a dislocation of the liver. Hydatids, abscess, and cancer were excluded, from absence of symptoms peculiar to each, although the indefinite cause of the father's death made me consider cancer carefully. The knowledge of the patient's habit of tippling, which I learned after her death, would have confirmed my suspicion of cirrhosis. Before the symptoms of the second stage of cirrhosis developed it was only suspected; for in the first stage the liver is enlarged uniformly; this was of one lobe. The symptoms of the second stage confirmed my suspicion, although there was no contraction.

The commencing Bright's disease was aggravated by ascites, no doubt. The amount of fluid was never so great as to interfere with respiration or cardiac action. The state of the mind forbade tapping.

The cause of death was undoubtedly softening of the brain. The renal complication was not sufficiently grave to cause uræmia. The cirrhotic cachexia was not sufficiently developed to produce the cerebral symptoms of the later stage of cirrhosis.—*Medical Times.*

